# Supplementary material for: The causal relationship between the human gut microbiota and pyogenic arthritis: a Mendelian randomization study
Source: Front Cell Infect Microbiol. 2024 Nov 26;14:1452480. doi: 10.3389/fcimb.2024.1452480 (PMC11629706; doi:10.3389/fcimb.2024.1452480)
Supplement: Supplementary file 1 [file DataSheet1.zip › mendelian test/ebi-a-GCST90027813.csv_forest.pdf]

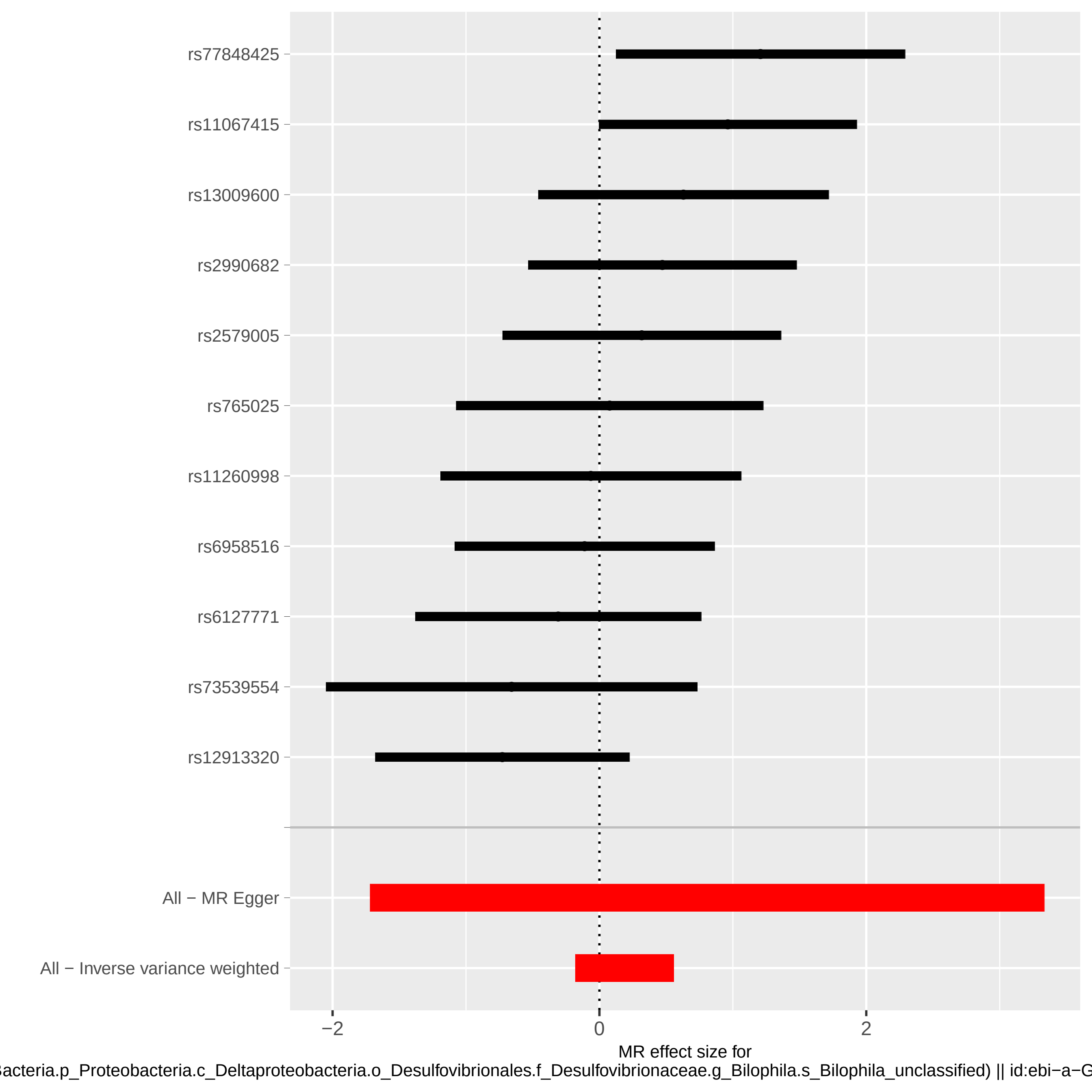
rs77848425
rs11067415
rs13009600
rs2990682
rs2579005
rs765025
rs11260998
rs6958516
rs6127771
rs73539554
rs12913320
All − MR Egger
All − Inverse variance weighted
−2
0
2
MR effect size for
Bacteria.p_Proteobacteria.c_Deltaproteobacteria.o_Desulfovibrionales.f_Desulfovibrionaceae.g_Bilophila.s_Bilophila_unclassified) || id:ebi−a−G
